# The new role of abortion in the context of low fertility in Czechia and Slovakia

**DOI:** 10.1038/s41598-026-54962-7

**Published:** 2026-06-08

**Authors:** Bára Idlbeková, Jiřina Kocourková

**Affiliations:** Department of Demography and Geodemography, Faculty of Science, Charles University, Prague, Czechia

**Keywords:** Health care, Medical research

## Abstract

This study examines how the role of abortion has evolved in the context of low fertility and delayed childbearing, with a focus on Czechia and Slovakia—two countries that experienced both high abortion rates and a demographic shift toward later motherhood since the 1990s. Using demographic data and survey analysis, it investigates the interplay between abortion, fertility, and contraceptive use under changing reproductive regimes. The analysis is structured around three objectives: (1) to assess long-term changes in the intensity and structure of induced and spontaneous abortions since 1993; (2) to evaluate the relationship between modern contraceptive use and abortion trends; and (3) to compare evolving public attitudes toward abortion in both countries. Data from national statistical offices were used for the demographic component, while binary logistic regression was applied to European Values Study data to explore the sociocultural dimension. The findings show that in low-fertility contexts with liberal abortion laws and widespread contraceptive access, abortion rates can remain low even as contraceptive prevalence declines, suggesting a weakening of the direct association between contraceptive use and abortion trends. This challenges the assumption that abortion levels directly reflect contraceptive failure. The study offers new theoretical insights into how reproductive behavior adapts to late fertility patterns and highlights the need for nuanced reproductive health policies that account for shifting social norms and individual agency.

## Introduction

Abortion is an important component of demographic processes, as high abortion rates can lead to significant reproductive losses with broader social implications^1^. In particular, the frequency of terminations of pregnancy (induced abortions) serves as a key public health indicator^2,3^. The prevalence of induced abortion is closely tied not only to the existence of liberal legislation and societal attitudes toward abortion but also to the availability and promotion of modern contraceptive methods^4^. Advances in contraceptive technologies and their widespread accessibility have had a profound effect not only on abortion rates^5^ but also on fertility levels and trends^6^.

The role of abortion can shift in response to changes in reproductive regimes. A major trend in recent decades in developed countries has been the postponement of childbearing to later maternal ages^7–9^. This trend began in the 1990s in Eastern European countries, where abortion rates had historically been high^10^. In some of these countries, it was not uncommon for women to have experienced three or more induced abortions^11,12^. The transformation of reproductive regimes across this region progressed at varying speeds and was accompanied by declining fertility, often falling to around 1.3 children per woman, well below replacement level by the late 1990s^13^. Abortion rates also generally declined during the 1990s, although some countries (e.g., Romania) experienced temporary fluctuations^14^. By the end of the 1990s, most Eastern European countries had significantly lower abortion rates than in the late 1980s, primarily due to the spread of effective contraception^15^. Notably, unlike in Western European countries such as Sweden and France, the shift toward later childbearing in Eastern Europe did not lead to a rise in abortion rates^16,17^. In Western European context, early phases of fertility postponement were often associated with increased exposure to unintended pregnancies before stable family formation, which contributed to temporarily higher abortion rates during the transition to later childbearing.

Within Eastern Europe, Czechia and Slovakia present unique cases due to their specific histories regarding abortion and contraceptive use, making them ideal for a comparative analysis of abortion trends in the context of changing reproductive regimes. These two countries share the advantage of complete and harmonized abortion statistics dating back to 1957. Underreporting, a common challenge in abortion statistics worldwide^15^, is not a major issue here because abortion has long been widely available and carries little social stigma. Before 1993, Czechia and Slovakia were part of Czechoslovakia, sharing identical legislation that allowed induced abortion on request up to 12 weeks of gestation. Since 1993, both countries have retained this liberal legal framework^18^. These conditions provide a unique opportunity to explore the evolving relationship between abortion and fertility in a setting of long-standing liberal abortion laws.

Over the past three decades, reproductive behaviour in Czechia and Slovakia has undergone profound and dynamic changes shaped by new social, economic, cultural, and political conditions. Both countries transitioned from early to late fertility regimes and moved from the group of countries with high abortion rates to those with relatively low rates^15,18^. Slovakia, in particular, experienced one of the steepest declines in abortion rates, alongside Estonia, Norway, and Slovenia^18^. While many post-1993 developments have been similar, distinct differences remain between the two populations^19^. In recent decades, European societies have experienced a broader transformation of reproductive behavior characterized by sustained low fertility, delayed childbearing, widespread use of effective contraception, and changing partnership trajectories. These shifts have given rise to new reproductive regimes in which reproduction is increasingly planned, intentional, and closely linked to individual life-course strategies. A detailed comparative analysis is essential to understand how the role of abortion has evolved within these new reproductive regimes.

This study has three objectives. First, we assess how the intensity and structure of induced and spontaneous abortions changed after 1993 in relation to declining fertility and the emergence of a late fertility model, and whether convergence in abortion behaviour continued between Czechia and Slovakia^18^. Second, we examine trends in modern contraceptive use and its impact on abortion and fertility rates, and to determine whether contraceptive practices in the two countries converged after 2008. Third, given the documented differences in abortion attitudes, particularly Slovakia’s lower tolerance for abortion due to its higher proportion of Catholic adherents^18^, the study evaluates whether societal attitudes are reflected in the evolving role of abortion within a low-fertility context.

## Background

Until the late 1980s, Czechia and Slovakia, then part of Czechoslovakia, belonged to the group of Central and Eastern European (CEE) countries for which the term “abortion culture” was used to describe the dominant birth-regulating behaviour^20,21^. At that time, the average Czech and Slovak woman experienced one induced abortion in her lifetime^18^, while in other countries such as the Soviet Union and Romania, it was not uncommon for women to undergo three to five abortions^11,12^. The concept of abortion culture explained high abortion rates not only through legal accessibility but also through liberal societal attitudes. Induced abortions were widely available upon request, mostly free of charge^20^, and socially accepted. They became a morally legitimate element of reproductive culture and were often viewed as part of women’s socialist emancipation^20,22^.

One key reason for high abortion rates in Czechoslovakia, as in other CEE countries, was the early adoption of liberal abortion laws in 1958, long before modern contraceptive methods such as IUDs and pills became widely accessible in the late 1960s^6^. In contrast, in Western Europe, the spread of modern contraception preceded the legalization of abortion^23^. While abortion became legally available in 1958 for “social reasons,” implementation was restrictive due to the requirement for approval by special committees. It was only with the 1987 reform, which abolished these committees, that women gained full autonomy in deciding the timing and number of their children. This liberal framework, allowing abortion on request up to 12 weeks, remained in force in both Czechia and Slovakia after their separation in 1993^18^.

Despite this liberal legislation, recent decades have seen diverging political and social trends in the two countries. In Slovakia, conservative political parties and the Catholic Church have pushed repeatedly for stricter abortion laws, reflecting more religious societal attitudes^18^. Since 2009, informed consent regulations require a 48-h waiting period after counseling on abortion’s risks^24^, and physicians may invoke conscientious objection to refuse performing abortions. Several legislative proposals to further restrict access, including doubling the waiting period and requiring women to state reasons for seeking abortion, have been repeatedly raised in political and parliamentary debate in recent years, though none have been enacted (2019–2021). In Czechia, in contrast, abortion access has faced little political challenge, and activism has remained limited to NGOs such as Hnutí pro život.

Abortion levels are shaped not only by liberal legislation but also by contraceptive practices, specifically the availability, use, and promotion of modern contraception^5,23,25^. In Western Europe, effective contraceptive methods became widespread in the 1970s and 1980s. In contrast, in CEE countries, traditional or no contraceptive methods predominated for decades due to limited access to modern contraception^11,26^. Although Czechoslovakia began producing contraceptive pills as early as 1966, they were difficult to obtain and had considerable side effects. Negative attitudes toward modern contraception persisted, and withdrawal remained the most common method until the early 1990s^6^. The earlier legalization of abortion before the availability of modern contraception contributed not only to high abortion rates but also to delayed acceptance of hormonal contraception in Czech and Slovak societies compared to Western Europe^6^.

After 1990, Western pharmaceutical companies introduced a range of safer hormonal contraceptives to CEE markets, creating favorable conditions for their wider use^11,27^. However, only Czechia experienced a substantial increase in modern contraceptive use during the 1990s^1,27^. In many post-Soviet states, induced abortion remained a common fertility regulation practice despite improved contraceptive access^12,28^. Barriers included cultural resistance, lack of preparedness to adopt modern methods, and skepticism among physicians regarding hormonal contraception, which reinforced perceptions of it as unnatural and harmful^29^.

Interestingly, in recent years, Western Europe has seen rising rejection of hormonal contraception, driven in part by social media platforms where women share negative experiences with contraceptive pills^30^. Reasons for rejecting hormonal contraception include side effects, concerns about mental health, sexuality, future fertility, and a growing preference for “natural” methods^31^.

In CEE, the 1990s marked the onset of the second demographic transition, characterized by fertility decline to sub-replacement levels and broader shifts in partnership and reproductive behaviour, including the postponement of childbearing to later maternal ages^9,13^. Both Czechia and Slovakia experienced a sharp fertility drop to extremely low levels (below 1.3 children per woman) in the 1990s, followed by slow recovery after 2000 driven by delayed childbearing and the gradual formation of late fertility regimes^19^.

The relationship between abortion, contraceptive use, and fertility trends has been widely studied^5,23,25^, but findings remain inconsistent. As Marston and Cleland argue, rising contraceptive use does not always reduce abortion rates, particularly when fertility declines rapidly^25^. This was evident in parts of CEE in the 1990s^6^. In high-fertility contexts, abortion often serves as a means of regulating unwanted births and maintaining early fertility patterns. In contrast, the transition to low fertility and late childbearing regimes raises new questions: how has the role of abortion evolved in Czechia and Slovakia in recent decades, and to what extent do contraception use and societal attitudes shape contemporary abortion levels?

## Data and methods

The demographic analysis of fertility and abortion was based on data published by the Czech Statistical Office (2023) and Statistical Office of the Slovak Republic (2023). At the time of manuscript preparation, 2022 represented the most recent year with harmonized and fully validated data available for both countries. Both countries possess long-term, complete records of abortion statistics dating back to their union as Czechoslovakia and continuing after their separation in 1993^32^. Reliable data on legal abortions are available from 1958, when abortion legislation was introduced alongside mandatory reporting by hospitals and physicians. The analysis was conducted over two periods: the first covering the years before separation (1980–1992) and the second after separation (1993–2022), allowing a comparison of trends within and between the two countries. In both Czechia and Slovakia, all types of abortions are registered, including spontaneous abortions, induced abortions (by all methods), and terminations of ectopic pregnancies. For brevity, the term “abortion” in this study refers specifically to induced abortions.

Key demographic indicators examined included:

- Age-specific fertility, induced abortion, and spontaneous abortion rates (number of live births, induced abortions, and spontaneous abortions per 1000 women in each age group);
- Mean age of women at childbirth, induced abortion, and spontaneous abortion (calculated from age-specific rates);
- Total induced abortion rate (TIAR) and total spontaneous abortion rate (TSAR), defined as the average number of induced/spontaneous abortions a woman would have over her reproductive life based on age-specific rates of a given year;
- Total fertility rate (TFR), defined as the average number of live births per woman based on age-specific fertility rates of a given year;
- Proportion of induced abortions out of all pregnancy outcomes. For the purpose of this analysis, the total number of pregnancies was defined as the sum of live births, induced abortions, and spontaneous abortions.

We also analysed data on modern contraceptive use obtained from the Institute of Health Information and Statistics of the Czech Republic (2022) and the National Health Information Centre in Slovakia (2023), which have maintained central records of medically prescribed contraceptives for women aged 15–49 since 1985. Modern contraceptive methods included hormonal contraception (pills) and intrauterine devices (IUDs), both of which required medical prescriptions. Modern contraceptive prevalence was defined as the percentage of women aged 15–49 using pills or/and IUDs.

To explore societal attitudes toward abortion, we utilised data from the 2017 European Values Study (EVS, 2017), the most recent fifth wave of a survey conducted in 40 European countries since 1981. The EVS sample included 1,811 respondents from Czechia and 1,432 from Slovakia, all aged 18+. The survey examined values and attitudes on family life, childrearing, religiosity, tolerance toward minority groups, and post-materialist values.

Attitudes toward abortion were assessed using the question: “Please indicate for each of the following whether you think it can always be justified, never be justified, or something in between,” rated on a 10-point scale (1 = “never justified”; 10 = “always justified”). We defined a binary dependent variable, justification of abortion, coded as 1 for responses 9–10 and 0 for responses 1–8. The binary variable measuring justification of abortion was constructed using a strict cut-off, coding responses with values 9–10 on the original 10-point scale as agreement, and values 1–8 as non-agreement. This approach captures strong normative approval rather than moderate or ambivalent attitudes. Alternative cut-off points (e.g. 8–10) were tested, but they resulted in substantially different distributions between countries, particularly in Slovakia, suggesting cross-national differences in the interpretation of intermediate scale values. To ensure cross-country comparability and a conservative interpretation of approval, only values 9–10 were retained as indicating justification of abortion. Missing responses were coded as system missing in SPSS and excluded from the analyses using listwise deletion.

We included independent variables identified in prior studies as predictors of abortion attitudes, such as gender, age, education, religiosity^18,33,34^, and attitudes toward homosexuality. Variables were recoded as follows: age: four groups (18–29, 30–44, 45–59, 60+); education: low (primary, vocational, secondary without diploma), medium (secondary with diploma, post-secondary), and high (bachelor’s degree or higher); religiosity: binary variable based on self-identification from the EVS question “Do you consider yourself a religious person?”, recoded as 1 = religious (category 1) and 0 = non-religious (categories 2 and 3, including convinced atheists). System missing responses (e.g., − 2 “no answer”, − 5 “other missing”) were excluded from the analysis ; justification of homosexuality: recoded as 1 for responses 9–10 (justified) and 0 for responses 1–8 (not justified).

We compared the proportion of respondents justifying abortion between Czechia and Slovakia across these characteristics. Binary logistic regression was used to assess associations between selected variables and abortion justification. Models were fitted using the Enter method. Odds ratios (ORs) with significance levels (*p < 0.05, **p < 0.01, ***p < 0.001) are reported. Model fit was evaluated using appropriate diagnostic procedures. All analyses were performed using SPSS version 20.

## Results

**Figure 1:**
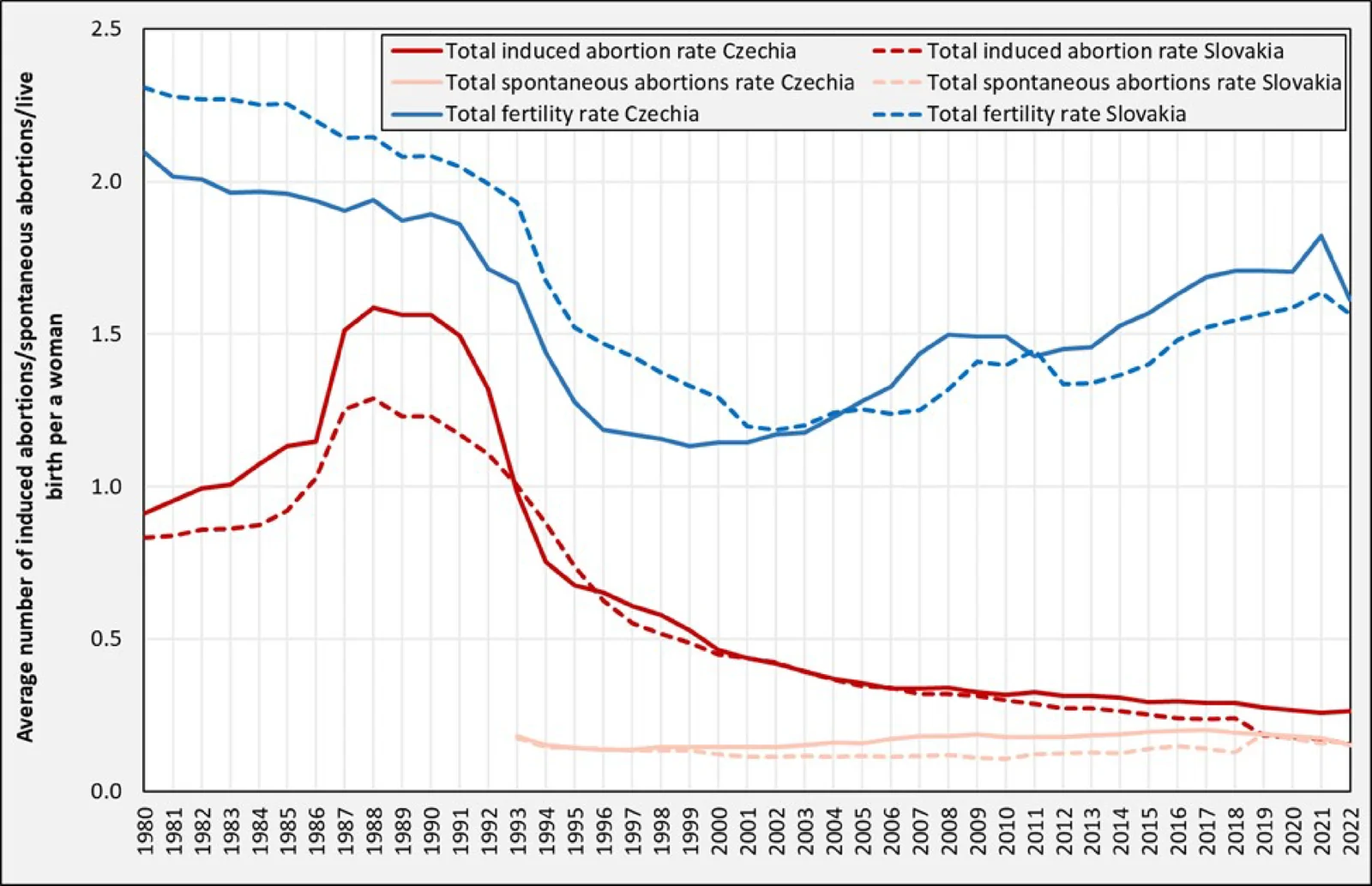
Trends in total induced abortion rate, total spontaneous abortion rate, and total fertility rate in Czechia and Slovakia, 1980–2022. *Data source*: Statistical Office of the Czech Republic, annual population and fertility statistics; Statistical Office of the Slovak Republic, annual population and fertility statistics.

Before 1990, within the common state of Czechoslovakia, Czechia and Slovakia exhibited notable differences in total induced abortion rates (TIAR) and total fertility rates (TFR) (Fig. 1). Following the liberalization of abortion legislation in 1987, there was a sharper increase in TIAR in Czechia, further widening the gap between the two regions. The highest TIAR was recorded in 1988, when Czech women averaged 1.6 induced abortions and Slovak women 1.3 induced abortions over their reproductive lifetime.

After 1990, Czechia experienced a steeper decline in TIAR, reaching lower values than Slovakia between 1993 and 1995. However, this trend reversed in 1996, when Slovakia’s decline in TIAR surpassed that of Czechia. By 2000, both countries had converged to the same level—below 0.5 induced abortions per woman—representing less than one-third of their 1988 values. This period of convergence continued until 2006. After 2007, divergent tendencies emerged: the decline in TIAR in Czechia slowed considerably, while in Slovakia it continued steadily. By 2022, the gap between the two countries had widened further, with Czechia’s TIAR reaching 0.26 and Slovakia’s dropping to just 0.15.

In contrast, total spontaneous abortion rates (TSAR) in both countries remained similar until the late 1990s (around 0.14). Divergence appeared after 2000, when TSAR in Czechia began to rise slightly, diverging from Slovakia’s values. However, from 2019 onwards, the rates in both countries converged again, reaching approximately 0.18 spontaneous abortions per woman.

After 1990, the trajectories of TIAR and TFR became independent of each other. In both countries, declines in TIAR were accompanied by simultaneous declines in TFR, albeit at different intensities. Czechia experienced a more rapid initial fertility decline, with TFR falling from 1.9 in 1990 to 1.1 in 1999, while Slovakia’s TFR decreased from 2.0 in 1990 to 1.3 in 1999. TFR values in both countries converged after 2000; however, from 2005 onwards, Czechia saw a faster increase in TFR, leading again to divergent patterns. Notably, both countries reached their respective TFR peaks in 2021, though at different levels—1.83 in Czechia and 1.64 in Slovakia.

These findings demonstrate that while Czechia and Slovakia shared broadly similar trends in TIAR, TSAR, and TFR over the past three decades, convergence was most evident during the brief period after 2000. Since 2006, divergent trajectories have predominated in both induced abortion and fertility patterns.

**Figure 2:**
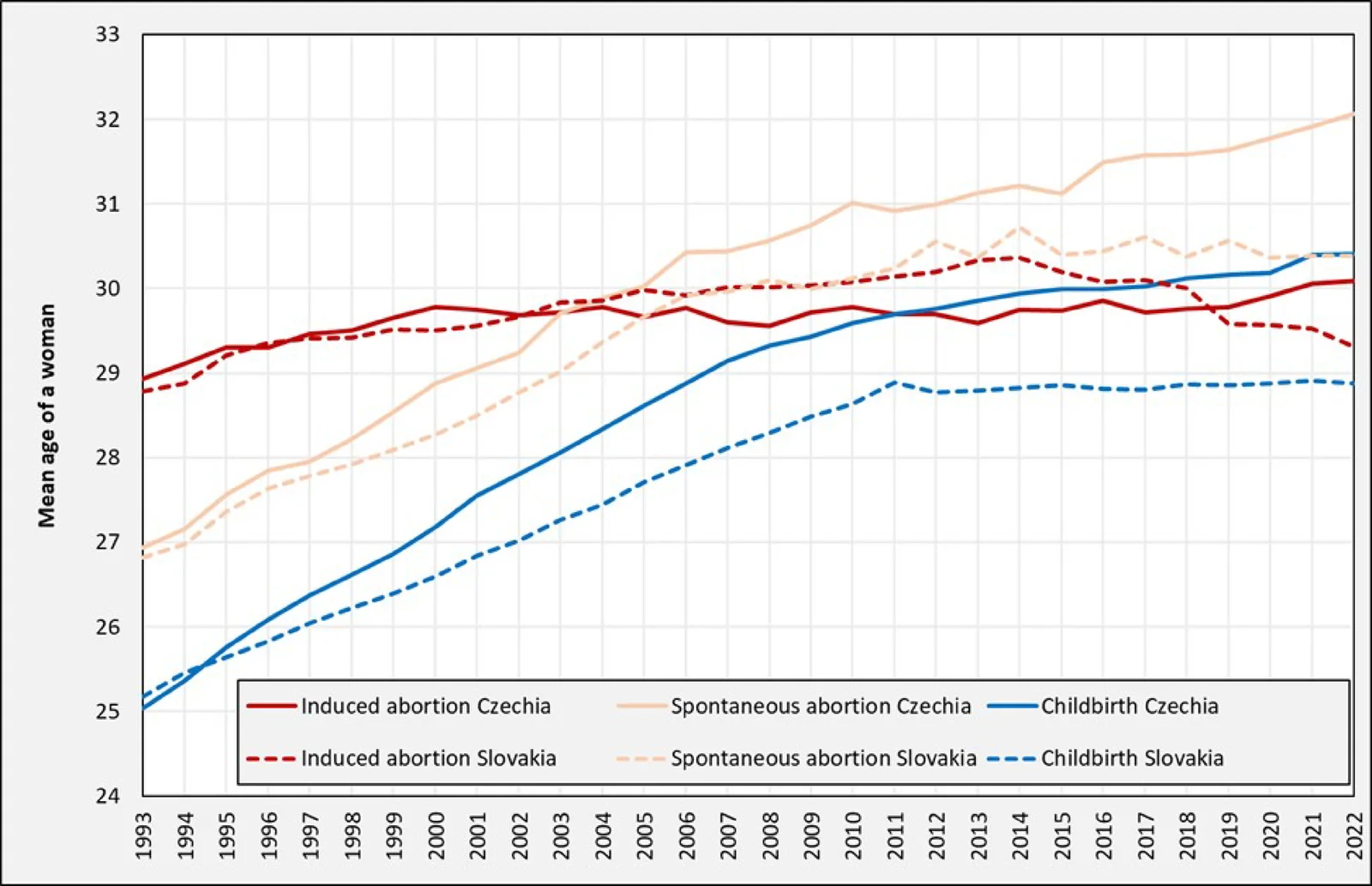
Trends in mean age of a woman at induced abortion, spontaneous abortion, and at childbirth in Czechia and Slovakia, 1993–2022. *Data source*: Statistical Office of the Czech Republic, annual population and fertility statistics; Statistical Office of the Slovak Republic, annual population and fertility statistics.

Trends in the timing of fertility and abortion also followed broadly similar patterns in both countries (Fig. 2). At the beginning of the observation period, the two populations exhibited comparable mean ages: 29 years at induced abortion, 27 years at spontaneous abortion, and 25 years at childbirth.

Despite these initial similarities, subsequent developments associated with the postponement of childbearing to later ages progressed more rapidly in Czechia. This is evident in the steeper rise in both the mean age at spontaneous abortion and at childbirth, indicating that the transition to a late fertility regime occurred earlier and more intensively in Czechia. By 2022, this divergent development resulted in a 1.5-year gap between the two populations: in Czechia, the mean age at spontaneous abortion had risen to 32 years, and the mean age at childbirth reached 30.5 years.

In contrast, the mean age at induced abortion showed the least divergence between the countries. In Czechia, it increased only modestly, from 29 years in 1993 to 30 years by 2022. In Slovakia, a similar rise occurred a decade earlier, but this was followed by a decline, bringing the mean age at induced abortion back to 29 years by 2022. This reversal in Slovakia mirrors the divergence observed in the timing of fertility.

**Figure 3:**
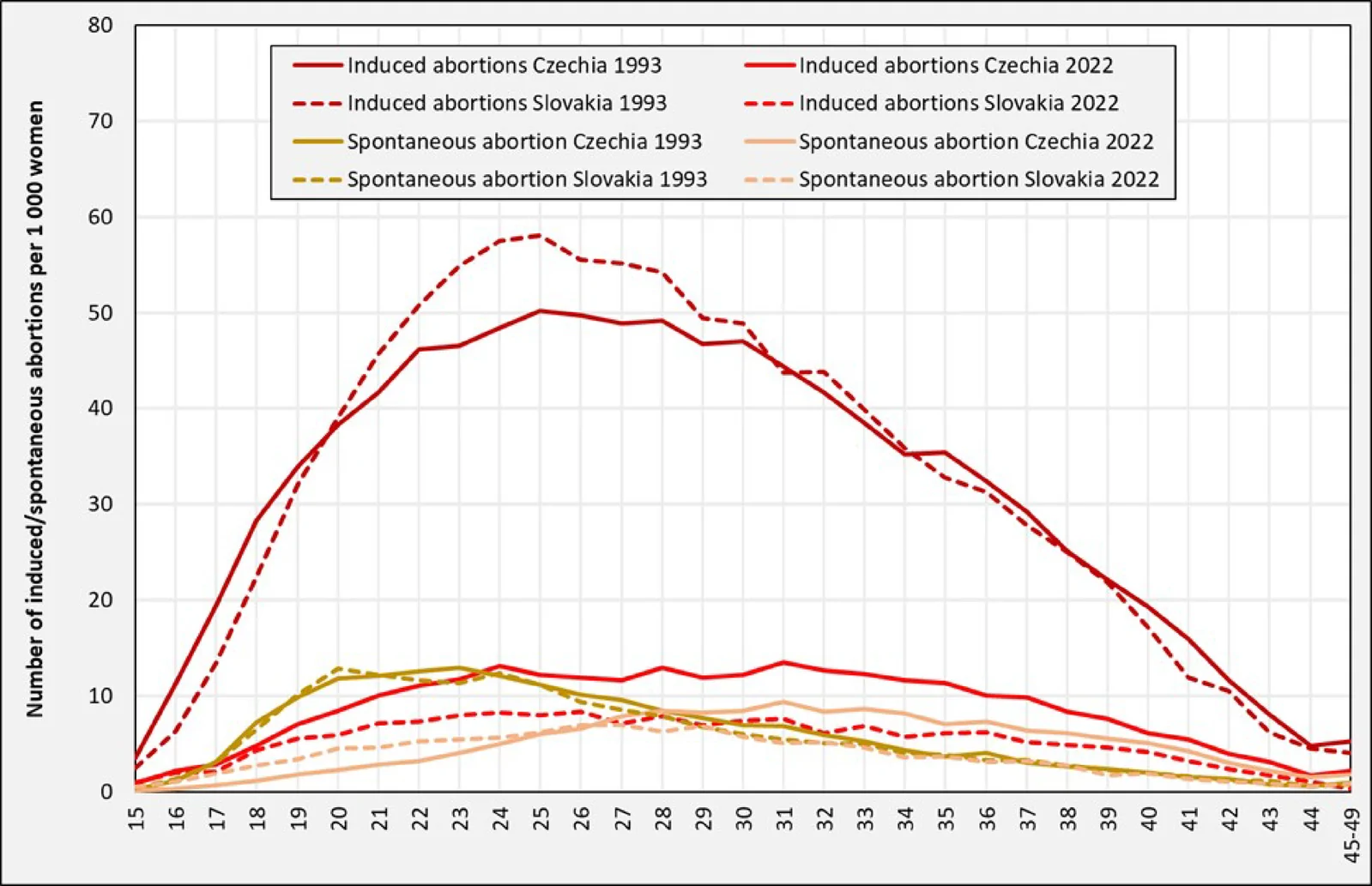
Age-specific induced abortion rates and spontaneous abortion rates in Czechia and Slovakia in 1993 and 2022. *Data source*: Statistical Office of the Czech Republic, annual population and fertility statistics; Statistical Office of the Slovak Republic, annual population and fertility statistics.

The development of age-specific induced abortion rates in Czechia and Slovakia highlights both a substantial overall decline across all age groups and a shift in the age distribution of women undergoing abortions (Fig. 3). In 1993, when abortion rates were already beginning to decline in both countries, the highest values were recorded among women aged 20–30. By the end of the observation period, rates had fallen significantly across all ages and were relatively evenly distributed among women aged 20–40, although slightly higher in Czechia.

In contrast, the age-specific spontaneous abortion rates followed a different trajectory. The peak shifted from the younger age group (20–25 years) to an older age group (30–35 years) in both countries. This shift reflects the transition to a late fertility regime, as advancing maternal age is associated with an increased risk of spontaneous abortion.

**Figure 4:**
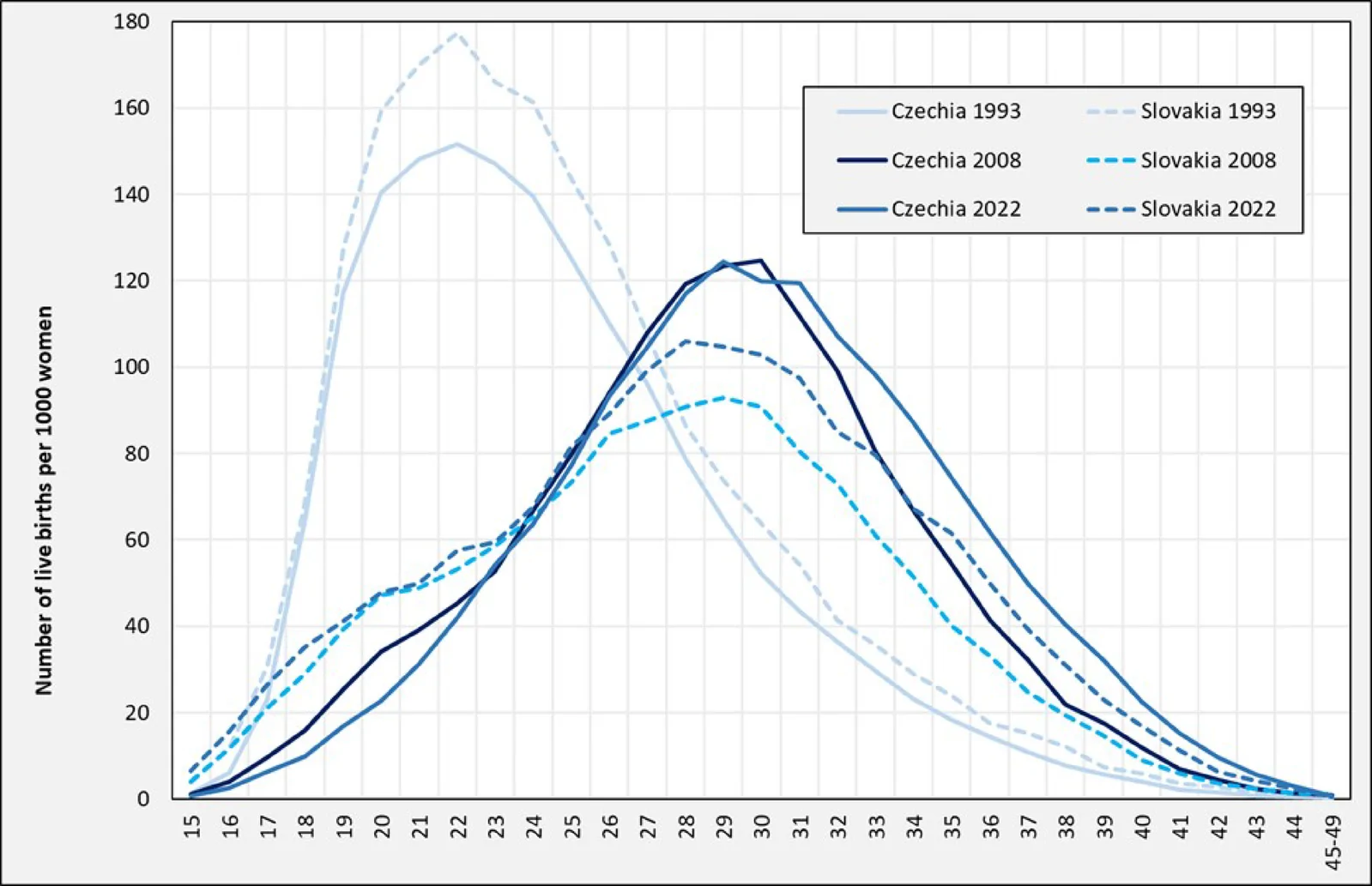
Age-specific fertility rates in Czechia and Slovakia in 1993, 2008 and 2022. *Data source*: Statistical Office of the Czech Republic, annual population and fertility statistics; Statistical Office of the Slovak Republic, annual population and fertility statistics.

Significant changes occurred in the distribution of age-specific fertility rates in both countries, marked by a shift in the peak of the fertility curve from age 22 in 1993 to age 28 in Slovakia and age 29 in Czechia (Fig. 4). The transformation of the fertility curves followed a similar pattern in both countries: the narrow concentration of fertility rates at the beginning of the reproductive period gave way to a broader distribution across the entire reproductive span.

However, in Slovakia, fertility rates in 2022 remained considerably higher among women up to age 23 and substantially lower at age 28 and older compared to Czechia. This pattern indicates a less pronounced transition to a late reproductive regime in Slovakia. Moreover, the transition to later childbearing was slower in Slovakia, as illustrated by the ASFR distribution in 2008: in Czechia, the late reproductive regime was already well established, whereas in Slovakia, it was still in the process of formation.

**Figure 5:**
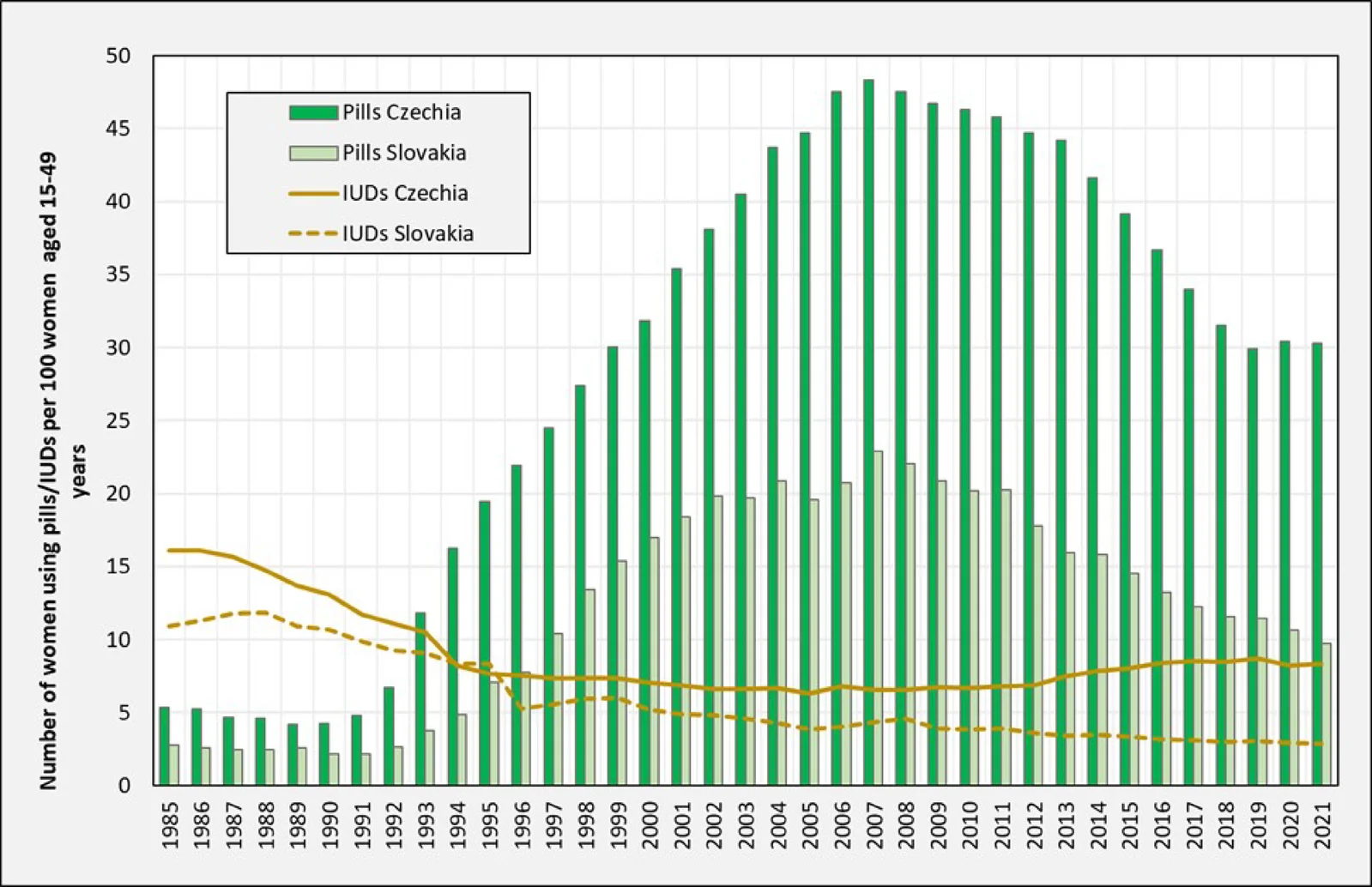
Trends in prevalence of medically prescribed contraceptives, pills and IUDs (in %) in Czechia and Slovakia, 1985–2021. *Data source*: Institute of Health Information and Statistics of the Czech Republic. Potraty 2021, National Health Information Centre of the Slovak Republic. Ženy užívajúce antikoncepciu na území SR za obdobie rokov 1991–2022.

The prevalence of contraception is a crucial factor in assessing overall trends in induced abortion. Figure 5 illustrates the development of medically prescribed contraceptive use, specifically hormonal contraception (pills) and intrauterine devices (IUDs). It is evident that these two methods followed markedly different trajectories throughout the observation period.

Before 1993, the prevalence of IUD use was higher in both countries compared to pills, which remained at very low levels (approximately 2.5% in Slovakia and 5% in Czechia). Subsequently, IUD use gradually declined as women increasingly turned to pills—this shift began in Czechia as early as 1993 and in Slovakia slightly later, in 1996. The prevalence of hormonal contraception then rose rapidly in both countries. The absolute peak was reached in 2007: in Czechia, 48% of women of reproductive age were using hormonal contraception, while Slovakia reached its maximum of 23% in the same year. This indicates that Slovakia lagged significantly behind Czechia, with a rate less than half that of its neighbor.

Following this peak, both countries experienced a continuous decline in hormonal contraception use. In Czechia, the decline halted in 2019 at 30%, whereas in Slovakia it continued slightly, reaching 10%. Despite broadly similar trends, the gap between the two countries widened, resulting in divergent trajectories: hormonal contraception use in Slovakia is now approximately three times lower than in Czechia.

The trends in IUD use followed a different pattern. Its importance diminished over time, but Czechia saw a modest resurgence after 2012, further increasing the differences between the two countries. Currently, in both Czechia and Slovakia, the prevalence of IUD use represents about one-third of the prevalence of pills.

**Figure 6:**
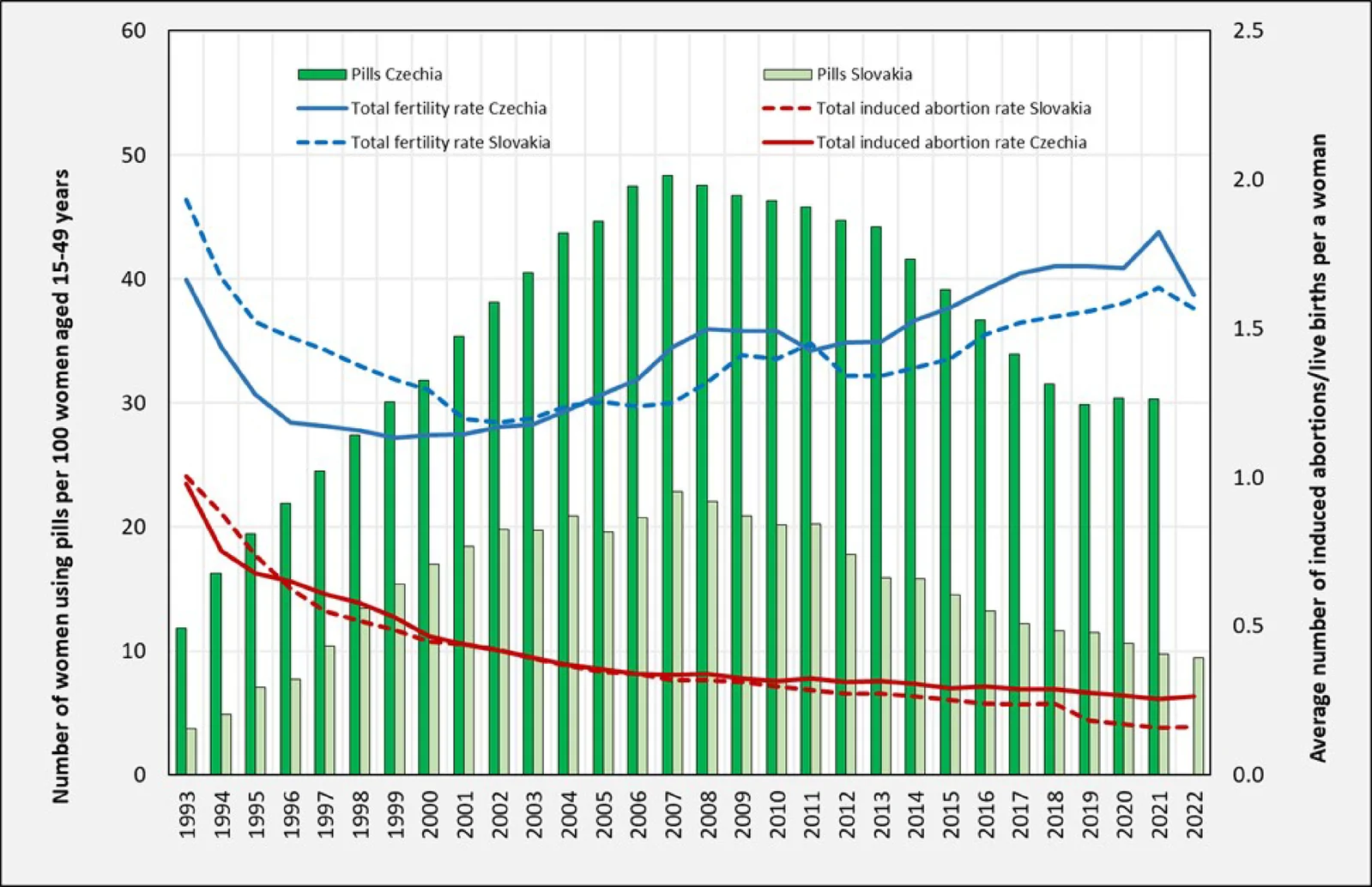
Trends in total induced abortion rate, total fertility rate, and contraceptive use (pills) in Czechia and Slovakia, 1993–2022. *Data source*: Statistical Office of the Czech Republic, annual population and fertility statistics; Statistical Office of the Slovak Republic, annual population and fertility statistics.

Figure 6 shows that while the development of the total induced abortion rate (TIAR) and the total fertility rate (TFR) has been independent of each other in both countries since 1993, the trend in TIAR has mirrored changes in the prevalence of pill use, particularly during the 1990s and early 2000s. Specifically, the decline in TIAR was accompanied by an increase in the use of pills. Although both countries experienced a parallel decline in TIAR and TFR, significant improvements in contraceptive behavior were observed only in Czechia. In contrast, Slovakia achieved low fertility levels with a much lower reliance on modern contraceptive methods.

After 2008, both countries experienced a turning point: the continued slight decline in TIAR was no longer accompanied by an increase in hormonal contraception use but rather by its decline. At the same time, TFR began to rise more markedly. This suggests that the decline in hormonal contraception use can partly be explained by the increase in TFR. Women who had begun using hormonal contraception intensively in the 1990s to postpone childbearing to later ages gradually entered the life stage after 2000 when they were starting families and therefore discontinued contraceptive use.

**Figure 7:**
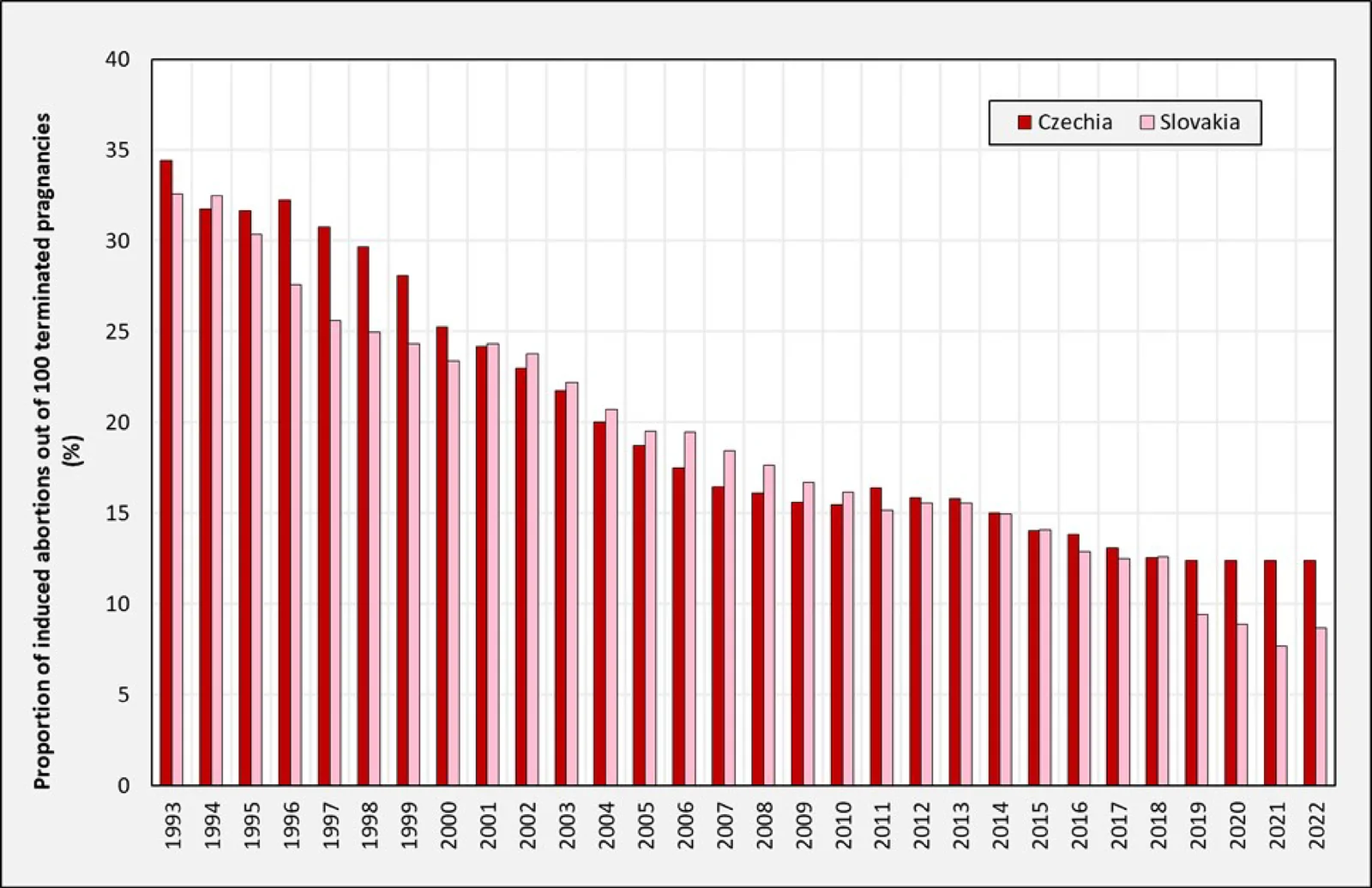
Trends in proportion of induced abortions out of all pregnancy outcomes. *Data source*: Statistical Office of the Czech Republic, annual population and fertility statistics; Statistical Office of the Slovak Republic, annual population and fertility statistics.

Figure 7 summarizes the overall effect of changes in reproductive and abortion behavior, highlighting a substantial decline in the proportion of pregnancies terminated by induced abortion in both countries. In 1993, nearly 35% of pregnancies in Czechia and 32% in Slovakia ended in abortion. By 2022, this proportion had decreased significantly to 13% in Czechia and 8% in Slovakia. The two countries came closest to convergence between 2012 and 2018. However, from 2019 onwards, the differences between them began to widen again.

Beyond demographic trends, differences in abortion patterns are also reflected in societal attitudes toward abortion. The evaluation of attitudes toward abortion based on the European Values Study (EVS) reveals substantial differences between Czechia and Slovakia (Table 1). The proportion of affirmative responses indicates that Czechia is considerably more tolerant of abortion justification than Slovakia (30% of men and 28% of women in Czechia versus 12% of men and 13% of women in Slovakia). The greatest differences are observed between Czech and Slovak men and among respondents aged 30–44 years.

**Table 1:** Attitudes towards abortion (proportion of justifications of abortion) in Czechia and Slovakia in 2017 by selected individual characteristics, and Odds ratios from Binary logistic regression analysis assessing associations between selected characteristics and justification of abortion.

	Czechia	Slovakia	Czechia	Slovakia	Czechia		Slovakia	
	Justification of abortion (%)	Justification of abortion (%)	Exp (B)	Exp (B)	95% C.I		95% C.I	
					Lower	Upper	Lower	Upper
Sex								
Men	30.3	12.0	1	1				
Women	27.8	12.7	0.679**	1.128	0.518	0.888	0.755	1.686
Age group								
18–29	33.0	19.7	1	1				
30–44	35.7	15.4	1.430	0.637	0.933	2.192	0.366	1.107
45–59	30.4	13.1	1.240	0.615	0.790	1.947	0.359	1.055
60+	21.8	7.1	1.116	0.428*	0.725	1.719	0.241	0.759
Education								
Low	21.3	7.2	1	1				
Medium	29.0	12.4	1.060	1.529	0.636	1.769	0.756	3.092
High	31.1	16.9	0.737	1.291	0.418	1.300	0.574	2.904
Justification of homosexuality								
Yes	54.6	50.6	9.008***	13.381***	6.810	11.914	9.036	19.815
No	11.9	6.5	1	1				
Religious person								
Yes	22.4	10.3	1	1				
No	33.1	20.7	1.477**	2.045***	1.115	1.956	1.362	3.071
Total %	28.8	12.5	–	–	–	–	–	–

Dependent variable is „justification of abortion “ having two values: 1 = answers 9 to 10 and 0 = answers 1 to 8 on the abortion justification scale (10 = I would always justify having an abortion, 1 = I would never justify having an abortion); Binary logistic regression, * < 0.05, ** < 0.01, *** < 0.001; number of respondents: Czechia (1,433), Slovakia (1,277); age of respondents is 18 + ; Nagelkerke R^2^: 28,7% Czechia, 29,8% Slovakia.

*Data source*: European Values Study. (2020). European Values Study 2017–2020 Integrated Dataset. GESIS Data Archive for the Social Sciences.

Binary logistic regression analysis, however, shows that gender has a significant influence on attitudes toward induced abortion only in Czechia, where men are more likely than women to justify abortion. In Slovakia, no significant gender effect was detected.

The relationship between age and abortion attitudes was not significant in Czechia across any age category. In Slovakia, a weak age effect was found in the 60 + age group, which exhibited lower tolerance for abortion compared to the youngest group (18–29 years). Overall, age appears to be an insignificant factor in shaping abortion attitudes in both countries.

Regarding education, no association with abortion views was found in either country. In contrast, religion and justification of homosexuality emerged as the strongest determinants of abortion attitudes. In Czechia, non-religious respondents were 1.5 times more likely to justify abortion than those identifying with a religion, while in Slovakia, this likelihood was twice as high. Support for homosexuality was also strongly associated with abortion tolerance: respondents in Czechia who justified homosexuality were nine times more likely to justify abortion, and in Slovakia, this likelihood was more than thirteen times higher.

## Discussion

### Fertility postponement and the transformation of abortion patterns

The aim of this article was to assess how the role of induced abortion has changed over the past three decades in the context of low fertility, using a comparative analysis of Czechia and Slovakia—countries that, until the late 1980s, ranked among those with high abortion rates and fertility close to replacement level. After 1993, both countries experienced a steep decline in total fertility rates (TFR) to 1.1 children per woman, followed by a gradual increase after 2000. This period was accompanied by a transition from an early to a late fertility regime^9,19^. In Czechia, this shift was largely completed by 2008, when the TFR reached its first peak of 1.5 children per woman. The transition in Slovakia was slower, and fertility rates among older women remain significantly lower than in Czechia.

It is noteworthy that until 2007, the prevalence of hormonal contraception increased, reaching its maximum in both countries (48% in Czechia and 23% in Slovakia), coinciding with a reduction in total induced abortion rates to the same level of 0.3 abortions per woman (from 1.0 in 1993). Importantly, this low (and slightly declining) abortion rate persisted in both countries until 2022, even as the use of hormonal contraception fell substantially after 2007 (to 30% in Czechia and 10% in Slovakia in 2022). These findings suggest that within a late fertility regime, it is possible to achieve and sustain a low level of induced abortion both at high and low prevalence of modern contraceptive methods. In the context of liberal abortion legislation and widespread contraceptive availability, our findings suggest that the direct association between contraceptive prevalence and abortion rates has weakened. Rather than indicating a complete decoupling, this pattern reflects a more complex relationship in which contraceptive prevalence alone no longer fully captures abortion dynamics in contemporary low-fertility societies.

### Reinterpreting the contraception–abortion relationship in low-fertility contexts

The present analysis is based on aggregate-level data and does not allow for causal inference regarding the relationship between contraceptive use and abortion rates. The observed parallel declines in contraceptive prevalence and abortion levels after 2008 coincide with rising fertility, which likely reflects intentional discontinuation of contraception in order to conceive rather than a weakening role of contraception in preventing unintended pregnancies. These trends should therefore be interpreted primarily as period effects associated with the transition to later childbearing and changing reproductive timing, rather than as evidence of a causal decoupling between contraception and abortion.

In addition, cohort effects and changes in sexual behavior may also contribute to these aggregate patterns. Younger cohorts entering reproductive ages differ from earlier cohorts in partnership formation, fertility intentions, and contraceptive practices, while changes in sexual activity over the life course and across cohorts may further affect the risk of unintended pregnancy. These processes cannot be disentangled using aggregate indicators but are important for understanding contemporary abortion dynamics.

These developments can be more explicitly situated within the framework of the Second Demographic Transition^13^. From this perspective, fertility postponement reflects both a driver and an outcome of broader shifts in values, partnership behavior, and reproductive decision-making. Tempo effects associated with delayed childbearing contribute to short- and medium-term fluctuations in fertility indicators, while quantum effects determine completed family size in the longer run. In this context, changes in abortion and contraceptive behavior should be understood as part of a wider reorganization of reproductive strategies accompanying the transition to later and more intentional childbearing, rather than as isolated responses to contraceptive availability alone.

In this context of delayed childbearing, it is also important to interpret the slight increase in spontaneous abortion observed at older maternal ages in Czechia, given the well-established association between advancing maternal age and pregnancy loss^35^. The risk of spontaneous abortion is strongly associated with increased rates of chromosomal abnormalities and pregnancy loss at older reproductive ages. As fertility is increasingly postponed, a larger share of pregnancies occurs at ages with elevated miscarriage risk, contributing to rising levels of spontaneous abortion. At the same time, improvements in early pregnancy detection and registration may lead to higher reported rates over time. Other contributing factors include underlying health conditions, lifestyle factors, and, to some extent, the growing use of assisted reproductive technologies^36^, which are associated with higher rates of early pregnancy loss. These processes should be taken into account when interpreting trends in spontaneous abortion in low-fertility societies.

### Diverging pathways: institutional, cultural, and behavioral explanations

Despite the steeper decline in contraceptive prevalence after 2007, Slovakia experienced a more significant decrease in total induced abortion rates (reaching 0.15 by 2022) than Czechia. This divergent trend may reflect intensified efforts to restrict access to abortion in Slovakia, resulting in reduced practical availability. Statistics on abortion reflect legislative, cultural, and religious influences on society^37^, which shape accessibility, potential delays in abortion services, and healthcare providers’ attitudes toward abortion^38^. In Slovakia, limited access may also result from the obligatory waiting period introduced in 2009, requiring a two-day delay between pre-abortion counseling and the procedure—similar to measures in Germany and Italy^37^. Furthermore, Slovak physicians can invoke a “conscientious objection” clause, allowing them to refuse to perform abortions on moral or religious grounds. In practice, entire healthcare facilities sometimes refuse to provide abortions, further restricting access compared to Czechia. Reduced access may also be linked to the fact that while the abortion pill (mifepristone) has been legally available in Czechia since 2014, its use remains limited to hospitals due to legal requirements that abortions be performed in medical facilities; in contrast, the pill remains unavailable under Slovak legislation.

In addition to religiosity, political context, and access to abortion services, several alternative mechanisms may contribute to recent abortion trends. These include increasing infertility at older reproductive ages, changes in union formation and the growing prevalence of cohabitation, shifts in sexual behavior among younger cohorts, and changes in the incidence of unintended pregnancies. The growing prevalence of cohabitation may also contribute to these trends, as cohabiting unions are typically less stable and less institutionalized than marriage. This can increase uncertainty surrounding childbearing decisions and, in the case of unintended pregnancies, raise the likelihood of pregnancy termination. Moreover, patterns of contraceptive discontinuation and switching may influence abortion risks independently of overall contraceptive prevalence. While these factors cannot be directly assessed using the available aggregate data, acknowledging their potential role is essential for a balanced interpretation of recent trends and cross-country differences.

In Western European countries, the postponement of childbearing into later ages began in the 1970s. In some cases, such as France and Sweden, this trend was associated with a temporary increase in abortion rates^16,17^. Postponing reproduction lengthens the period between sexual debut and first birth, increasing challenges in daily contraceptive use and raising the risk of unplanned pregnancies and higher teenage abortion rates. By contrast, in Czechia and Slovakia, the onset of delayed childbearing in the 1990s was accompanied by a sharp decline in abortion rates, even without any legislative changes. Fears of increased abortion rates due to poor pregnancy timing, as observed in Sweden and France^39^, were not realized. Instead, both populations recorded a significant reduction in abortion rates across all age groups. This trajectory diverged from prior trends in Western Europe and the persistent high abortion rates in other Eastern European countries^10^. Although similar declines in abortion rates were observed across other Eastern European countries during the 1990s, the decrease was more gradual and less clearly linked to the postponement of childbearing to older ages, as this postponement itself progressed more slowly^6^.

The rise in hormonal contraceptive use played a crucial role in the initial transformation of the fertility regime, ensuring that fertility declines were not accompanied by rising abortion rates. The Czech and Slovak examples confirm that establishing a new reproductive model characterized by delayed parenthood would have been delayed without a substantial increase in effective contraceptive prevalence^6^. In Slovakia, the rise in hormonal contraceptive use was less pronounced but still sufficient to contribute to the decline in abortion rates.

However, Slovakia has historically had lower abortion rates, likely due to higher religiosity^18^. This also helps explain why Slovak women have consistently lower contraceptive prevalence than Czech women. Despite parallel trends—increasing contraceptive prevalence until 2007 and subsequent decline thereafter—the gap between the two countries widened, with Slovakia’s hormonal contraceptive prevalence in 2022 returning to levels seen in the late 1990s. Recent declines in hormonal contraceptive use have also been observed in Western Europe. A growing hormone skepticism, amplified by social media platforms, has contributed to decreased use of effective contraception and, in some countries such as France, an increase in unplanned pregnancies and abortions^40^. Social media has played a key role in shaping perceptions of hormonal contraception, as women share negative experiences online^41^. Such sharing of personal narratives introduces a new dimension of knowledge, contrasting with abstract statistical risk data^42^. This phenomenon influences decision-making about contraceptive methods^43,44^ and has been linked to declining hormonal contraceptive use and the adoption of alternative contraceptive technologies in several Western European countries over the past decade^45^.

Even after the transition to a late fertility regime, differences in the intensity of delayed reproduction persist and are reflected in abortion patterns. In Slovakia, the rise in the mean age of mothers at childbirth plateaued in 2011 and has since stabilized at 29 years, while in Czechia, it continued to increase, reaching 30.3 years in 2022. This divergence is mirrored in the mean age of women at spontaneous abortion, which has continued to rise in Czechia (32 years) but stabilized in Slovakia (30.5 years). Divergence has also emerged in the timing of induced abortions. While abortion trends converged in both countries until 2008, divergent developments have since emerged. In Czechia, the decline in hormonal contraceptive use was not adequately offset by increased use of other effective methods (e.g., condoms), potentially contributing to a significant slowdown in the decline of abortion rates.

Although low abortion rates are often associated with high contraceptive prevalence^5^, the Czech and Slovak cases illustrate that other factors can also sustain low abortion rates despite reduced contraceptive use. These include higher education levels and greater economic stability, which support delayed childbearing and more deliberate reproductive planning, reducing the likelihood of unplanned pregnancies. However, education and age were not significant predictors of attitudes toward abortion in either country. Cultural and religious factors remain crucial, particularly in Slovakia^18^. Thus, in contemporary low-fertility societies, abortion levels are shaped by a broader set of factors beyond contraceptive prevalence alone. These include delayed and more deliberate reproductive decision-making, partnership stability, socio-economic security, access to reproductive health services, and prevailing cultural and religious norms. In this context, abortion increasingly functions as a residual mechanism addressing contraceptive failure or exceptional life circumstances rather than as a primary means of fertility regulation.

Both Czechia and Slovakia now rank among European countries with low abortion rates. This development marks a clear departure from the “abortion culture” of the pre-1993 period, when abortions were widely used as a means of birth control^18^. Nevertheless, in 2022, 12.5% of pregnancies in Czechia and 9% in Slovakia still ended in abortion. While contraceptive failures will likely sustain a certain level of abortion^5^, contraception and abortion remain as the principal means of birth regulation^11^.

Several limitations of this study should be acknowledged. First, our analysis of contraceptive behavior is limited to medically prescribed methods—hormonal contraception and intrauterine devices—for which reliable long-term data are available. Other methods, such as condoms or natural family planning, may play a non-negligible role in birth control, particularly in recent years, but could not be included due to the lack of comparable longitudinal data. In contrast to medically prescribed methods, condom use is primarily captured through survey-based data and varies substantially by age, partnership status, and life-course stage, which makes it difficult to incorporate into consistent long-term time series for both countries. Second, the analysis does not capture potential changes in the effectiveness of contraceptive use, including inconsistent or incorrect use of specific methods, nor changes in sexual activity over time. These factors may influence the risk of unintended pregnancy independently of overall contraceptive prevalence. Consequently, our findings were interpreted as evidence of a weakening, rather than a complete disappearance, of the association between contraceptive prevalence and abortion rates in the current low-fertility context.

Another limitation concerns potential changes in measurement and reporting over time. Although abortion registration in Czechia and Slovakia is generally considered highly complete and reliable, reporting practices may not have remained entirely constant across decades. In Czechia, the increasing use of medical abortion may affect the classification and timing of registered abortions, potentially influencing observed trends. In Slovakia, observed declines in abortion rates may partly reflect reduced access to abortion services, including institutional barriers and provider-level conscientious objection, rather than a pure decline in incidence. These factors were taken into account when cross-country differences and long-term trends in abortion levels were interpreted.

The regression analysis has additional limitations. The binary operationalization of abortion justification reduces attitudinal variation and may attenuate effect sizes, although it allows for a clearer interpretation of strong normative approval. While standard diagnostics indicated an adequate model fit, only key summary statistics are reported here given the descriptive and exploratory purpose of the analysis. Furthermore, some explanatory variables, particularly religiosity and attitudes toward homosexuality, are conceptually and empirically related, which may introduce multicollinearity. This potential overlap was considered when the magnitude of individual coefficients was interpreted.

In conclusion, the declining trend in abortion rates in both countries no longer appears directly linked to increasing hormonal contraceptive prevalence. The decline in contraceptive use likely reflects broader shifts in partnership patterns, sexual behavior, and the growing preference for “natural” health practices, which may conflict with the use of synthetic hormones and pharmaceuticals. Further research is needed to understand these changes in greater detail, particularly age-specific data on contraceptive use, which would help interpret these trends and identify potential risks for reproductive health policy. Future research would also benefit from a more detailed life-course perspective on abortion, focusing on women’s partnership status, parity, and the timing of abortion relative to birth order (e.g. before the first birth or between subsequent births). Such analyses would help to further elucidate how the role of abortion has changed within late fertility regimes and to distinguish between different reproductive trajectories.

## Data sources

- Czech Statistical Office. Demografické ročenky (pramenná díla) 1990–2022. Praha: Czech Statistical Office; 2023. Available from: https://www.czso.cz/csu/czso/casova_rada_demografie_2009_1990
- European Values Study 2017: Integrated Dataset. Cologne: GESIS Data Archive; 2020. Available from: https://europeanvaluesstudy.eu/methodology-data-documentation/survey-2017/full-release-evs2017/
- Institute of Health Information and Statistics of the Czech Republic. Potraty 2021. Praha: ÚZIS ČR; 2022. ISBN: 978-80-7472-172-4. Available from: https://www.uzis.cz/res/f/008422/potraty2021.pdf
- National Health Information Centre of the Slovak Republic. Ženy užívajúce antikoncepciu na území SR za obdobie rokov 1991–2022. Bratislava: NCZI; Available from: http://www.nczisk.sk

- Statistical Office of the Slovak Republic. Potraty 1993–2022. Bratislava: ŠÚ SR; 2023. Available from: https://slovak.statistics.sk

## Data Availability

All data analysed in this study are publicly available from the Czech Statistical Office, the Statistical Office of the Slovak Republic, and the European Values Study 2017 dataset.
